# Neural markers of predictive coding under perceptual uncertainty revealed with Hierarchical Frequency Tagging

**DOI:** 10.7554/eLife.22749

**Published:** 2017-02-28

**Authors:** Noam Gordon, Roger Koenig-Robert, Naotsugu Tsuchiya, Jeroen JA van Boxtel, Jakob Hohwy

**Affiliations:** 1 Cognition and Philosophy Lab, Philosophy Department, Monash University, Clayton, Australia; 2 School of Psychology, The University of New South Wales, Sydney, Australia; 3 Monash Institute of Cognitive and Clinical Neurosciences, Monash University, Clayton, Australia; 4 School of Psychological Sciences, Monash University, Clayton, Australia; University of Zurich and ETH Zurich, Switzerland

**Keywords:** Hierarchical Frequency Tagging, predictive coding, Semantic Wavelet-Induced Frequency Tagging, intermodulation, Human

## Abstract

There is a growing understanding that both top-down and bottom-up signals underlie perception. But it is not known how these signals integrate with each other and how this depends on the perceived stimuli’s predictability. ‘Predictive coding’ theories describe this integration in terms of how well top-down predictions fit with bottom-up sensory input. Identifying neural markers for such signal integration is therefore essential for the study of perception and predictive coding theories. To achieve this, we combined EEG methods that preferentially tag different levels in the visual hierarchy. Importantly, we examined intermodulation components as a measure of integration between these signals. Our results link the different signals to core aspects of predictive coding, and suggest that top-down predictions indeed integrate with bottom-up signals in a manner that is modulated by the predictability of the sensory input, providing evidence for predictive coding and opening new avenues to studying such interactions in perception.

**DOI:** http://dx.doi.org/10.7554/eLife.22749.001

## Introduction

Perception is increasingly being understood to arise by means of cortical integration of ‘bottom-up’ or sensory-driven signals and ‘top-down’ information. Prior experience, expectations and knowledge about the world allow for the formation of priors or hypotheses about the state of the external world (i.e., the causes of the sensory input) that help, via top-down signals, resolve ambiguity in bottom-up sensory signals. Such neuronal representations, or ‘state-units’ can then be optimised in light of new sensory input. Early models of neural processing implementing such a predictive coding framework explicitly incorporated prior knowledge of statistical regularities in the environment (Srinivasan et al., 1982). Contemporary accounts treat these ideas in terms of Bayesian inference and prediction error minimization (Rao and Ballard, 1999; Friston, 2005; Friston and Stephan, 2007; Hohwy, 2013; Clark, 2013).

That perception is essentially an inferential process is supported by many behavioural findings demonstrating the significant role of contextual information (Geisler and Kersten, 2002; Kersten et al., 2004; Kok and Lange, 2015; Weiss et al., 2002) and of top-down signals (Kok et al., 2012b, Pascual-Leone and Walsh, 2001; Ro et al., 2003; Vetter et al., 2014) in perception. Several studies additionally suggest different neural measures of feedforward and feedback signals (Hupe et al., 1998) primarily in terms of their characteristic oscillatory frequency bands (Bastos et al., 2015; Buschman and Miller, 2007; Fontolan et al., 2014; Mayer et al., 2016; Michalareas et al., 2016; Sherman et al., 2016; van Kerkoerle et al., 2014).

However, studying the neural basis of perception requires not only distinguishing between top-down and bottom-up signals but also examining the actual integration between such signals. This is particularly important for predictive coding, which hypothesizes such integration as a mechanism for prediction error minimization. According to predictive coding this mechanism is marked by the probabilistic properties of predictions and prediction errors such as the level of certainty or precision attributed to the predictions. Hence, the goals of this study were to simultaneously tag top-down and bottom-up signals, to identify a direct neural marker for the integration of these signals during visual perception and, further, to examine if, and how, such a marker is modulated by the strength of prior expectations.

In order to differentiate between top-down signals related to predictions, bottom-up signals related to the accumulation of sensory input, and the interaction between such signals, we developed the Hierarchical Frequency Tagging (HFT) paradigm in which two frequency tagging methods are combined in the visual domain in a hierarchical manner. To preferentially track top-down signals (i.e., putative prediction signals) we used semantic wavelet induced frequency tagging (SWIFT) that has been shown to constantly activate low-level visual areas while periodically engaging high-level visual areas (thus, selectively tagging the high-level visual areas; [Koenig-Robert and VanRullen, 2013; Koenig-Robert et al., 2015]). To simultaneously track bottom-up signals we used classic frequency tagging, or so called steady state visual evoked potentials (SSVEP) (Norcia et al., 2015; Vialatte et al., 2010). We combined the two methods by presenting SWIFT-modulated images at 1.3 HZ while modulating the global luminance of the stimulus at 10 Hz to elicit SSVEP (See Materials and methods for details). Critically, we hypothesized that intermodulation (IM) components would appear as a marker of integration between these differentially tagged signals.

Intermodulation is a common phenomenon manifesting in non-linear systems. When the input signal is comprised of more than one fundamental frequency (e.g., *F1* and *F2*) that interact within a non-linear system, the response output will show additional frequencies as linear combinations of the input frequencies (e.g., *f1* +*f2*, *f1 - f2*, etc.) (note that throughout the paper we denote stimulus frequencies with capital letters (e.g., *F1*) and response frequencies with small letters (e.g., *f1*)). Intermodulation components in EEG recordings have been used to study non-linear interactions in the visual system (Clynes, 1961; Regan and Regan, 1988; Zemon and Ratliff, 1984), with some recent applications for the study of high-level visual-object recognition systems (Boremanse et al., 2013; Gundlach and Müller, 2013; Zhang et al., 2011). Instead of tagging two ‘bottom-up’ signals, however, our paradigm was designed to enable the examination of the integration between both bottom-up *and* top-down inputs to the lower visual areas.

Optimal perceptual inference relies on our ability to take into account the statistical properties of the stimuli and the context in which they occur. One such property is expectation, which reflects the continuous process of probabilistic learning about what is possible or probable in the forthcoming sensory environment (Summerfield and Egner, 2009) and therefore plays a central role in predictive coding. Indeed, various studies have demonstrated the relationship between stimulus predictability and neural responses (Kok et al., 2012a, Todorovic et al., 2011). Accordingly, we hypothesised that manipulating the predictability, or, as we label it, the level of *certainty* about the stimuli would modulate the IM responses. Certainty was manipulated by changing the frequency of images in each trial; the more frequent the image is presented, the easier to successfully predict what the next stimulus will be.

From the viewpoint of Bayesian belief updating, belief updates occur by combining predictions derived from prior probabilities with sensory-driven data, resulting in prediction errors which are weighted by their relative precisions (Mathys et al., 2014). The certainty manipulation thus affected the precision of predictions such that higher certainty means higher prior precision and less weighting for the bottom-up prediction error. The precision of the stimuli themselves (e.g. the level of noise in the stimulus) did not vary across trials.

Overall, our aim was therefore to find not only neural markers for the integration of sensory-driven and prediction-driven signals, but also to examine how this process is modulated by certainty – a core element in the predictive coding framework.

## Results

**Figure 1.**
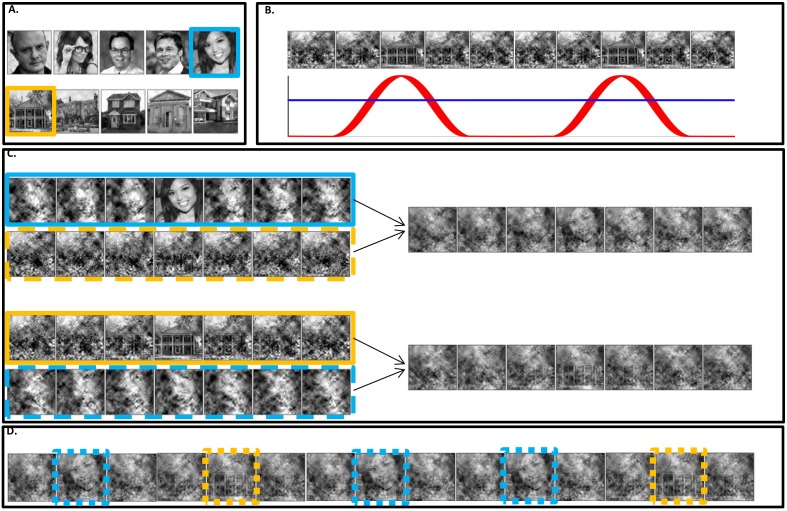
Stimuli construction. Schematic illustration of stimuli construction. (**A**) A pool of 28 face and 28 house images were used in the paradigm (images with ‘free to use, share or modify, even commercially’ usage rights, obtained from Google Images). (**B**) The SWIFT principle. Cyclic local-contour scrambling in the wavelet-domain allows us to modulate the semantics of the image at a given frequency (i.e. the tagging-frequency, F2 = 1.3 hz, illustrated by the red line) while keeping low-level principal physical attributes constant over time (illustrated by the blue line) (**C**) Each trial (50 s) was constructed using one SWIFT cycle (~769 ms) of a randomly chosen face image (blue solid rectangle) and one SWIFT cycle of a randomly chosen house image (orange solid rectangle). For each SWIFT cycle, a corresponding ‘noise’ SWIFT cycle was created based on one of the scrambled frames of the original SWIFT cycle (orange and blue dashed rectangles). Superimposition of the original (solid rectangles) and noise (dashed rectangles) SWIFT cycles ensures similar principal local physical properties across all SWIFT frames, regardless of the image appearing in each cycle. (**D**) The two SWIFT cycles (house and face) were presented repeatedly in a pseudo-random order for a total of 65 cycles. The resulting trial was a 50 s movie in which images peaked in a cyclic manner (F2 = 1.3 Hz). Finally, a global sinusoidal contrast modulation at F1 = 10 Hz was applied onto the whole movie to evoke the SSVEP. **DOI:** http://dx.doi.org/10.7554/eLife.22749.002

Participants were presented with 50 s ‘movie’ streams in which either a house or a face image appeared briefly at a frequency of 1.3 Hz (F2). Each 50 s trial was constructed using one face and one house image randomly selected from a pool of images. Images were scrambled using two frequency tagging methods - SWIFT and SSVEP - that differentially tag areas in the cortical hierarchy (Figure 1). Prior to each trial, participants were instructed to count the number of times one of the two images appeared in the trial (either the house or the face image) and they reported their response at the end of each trial. The proportion of images changed over trials, ranging from trials in which both images appeared in nearly half the cycles (referred to as ‘low certainty’ trials) to trials in which one of the images appeared in nearly all cycles (referred to as ‘high certainty’ trials).

**Figure 2.**
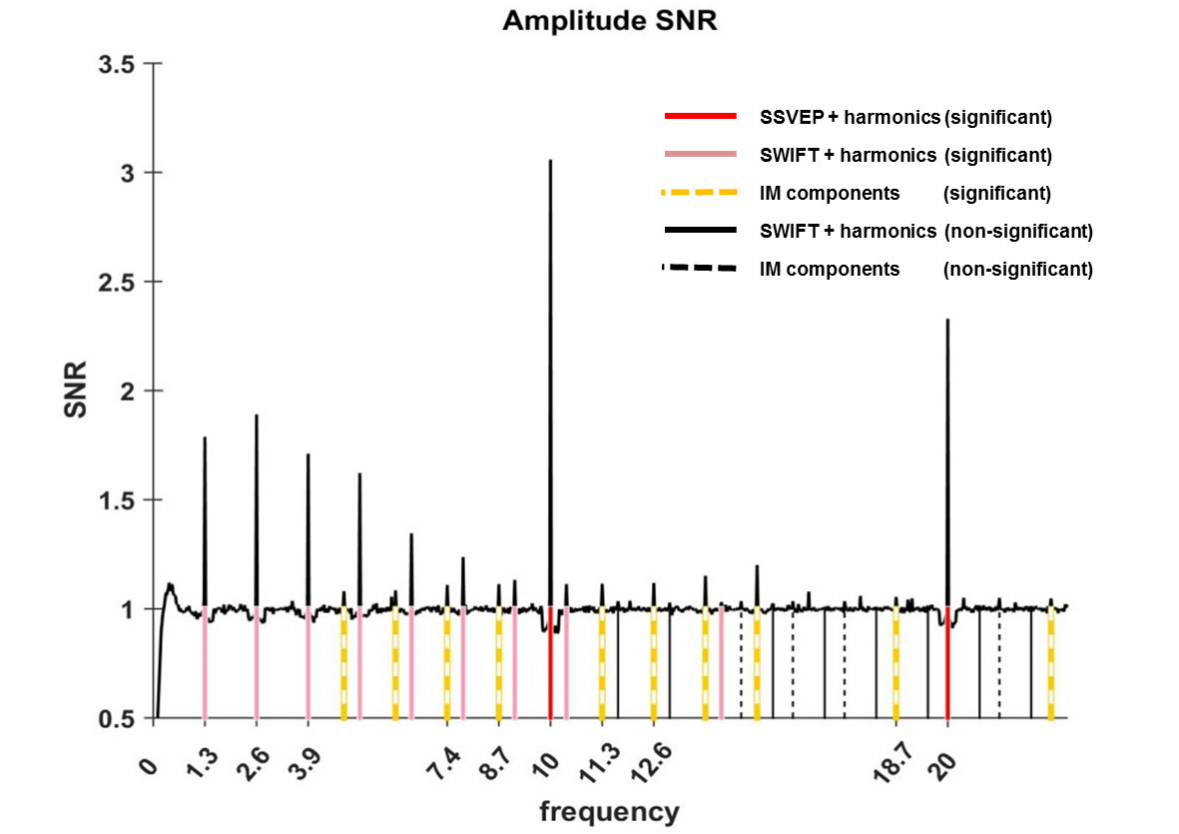
Amplitude SNR spectra. Amplitude SNRs (see Materials and methods for the definition of SNR), averaged across all electrodes, trials and participants, are shown for frequencies up to 23 Hz. Peaks can be seen at the tagging frequencies, their harmonics and at IM components. Solid red lines mark the SSVEP frequency and its harmonic (10 Hz and 20 Hz, both with SNRs significantly greater than one). Solid pink lines mark the SWIFT frequency and harmonics with SNRs significantly greater than one (n2f2 where n2 = 1,2,3…8 and 11). Solid black lines mark SWIFT harmonics with SNRs not significantly greater than one. Yellow dashed lines mark IM components with SNRs significantly greater than one (n1f1 + n2f2; n1 = 1, n2 = +−1,+−2,+−3,+−4 as well as n1 = 2, n2 = −1,+2) and black dashed lines mark IM components with SNRs not significantly greater than one. **DOI:** http://dx.doi.org/10.7554/eLife.22749.003

Having assured that participants were able to perform the task (Figure 6), we first verified whether our two frequency-tagging methods were indeed able to entrain brain activity, and whether we could observe intermodulation (IM) components. Figure 2 shows the results of the fast Fourier transform (FFT) averaged across all 64 electrodes, trials and participants (N = 17). Importantly, significant peaks can be seen at both tagging frequencies (f1 = 10 Hz and f2 = 1.3 Hz) and their harmonics (n1f1 and n2f2 where n1 = 1,2 and n2 = 1,2,3...8 and 11; red and pink solid lines in Figure 2) and at various IM components (n1f1 + n2f2 where n1 = 1, n2 = +−1,+−2,+−3,+−4 as well as n1 = 2, n2 = −1,+2; orange dashed lines in Figure 2) (one sample t-test, FDR-adjusted p<0.01 for frequencies of interest in the range of 1 Hz–40Hz).

After establishing that both tagging frequencies and their IM components are present in the data, we examined their spatial distribution on the scalp, averaged across all trials. We expected to find strongest SSVEP amplitudes over the occipital region (as the primary visual cortex is known to be a principal source of SSVEP [Di Russo et al., 2007]) and strongest SWIFT amplitudes over more temporal and parietal regions (as SWIFT has been shown to increasingly activate higher areas in the visual pathway [Koenig-Robert et al., 2015]). IM components, in contrast, should originate from local processing units which process both SSVEP and SWIFT inputs. Under the predictive coding framework, predictions are projected to lower levels in the cortical hierarchy where they are integrated with sensory input. We therefore speculated that IM signals will be found primarily over occipital regions.

**Figure 3.**
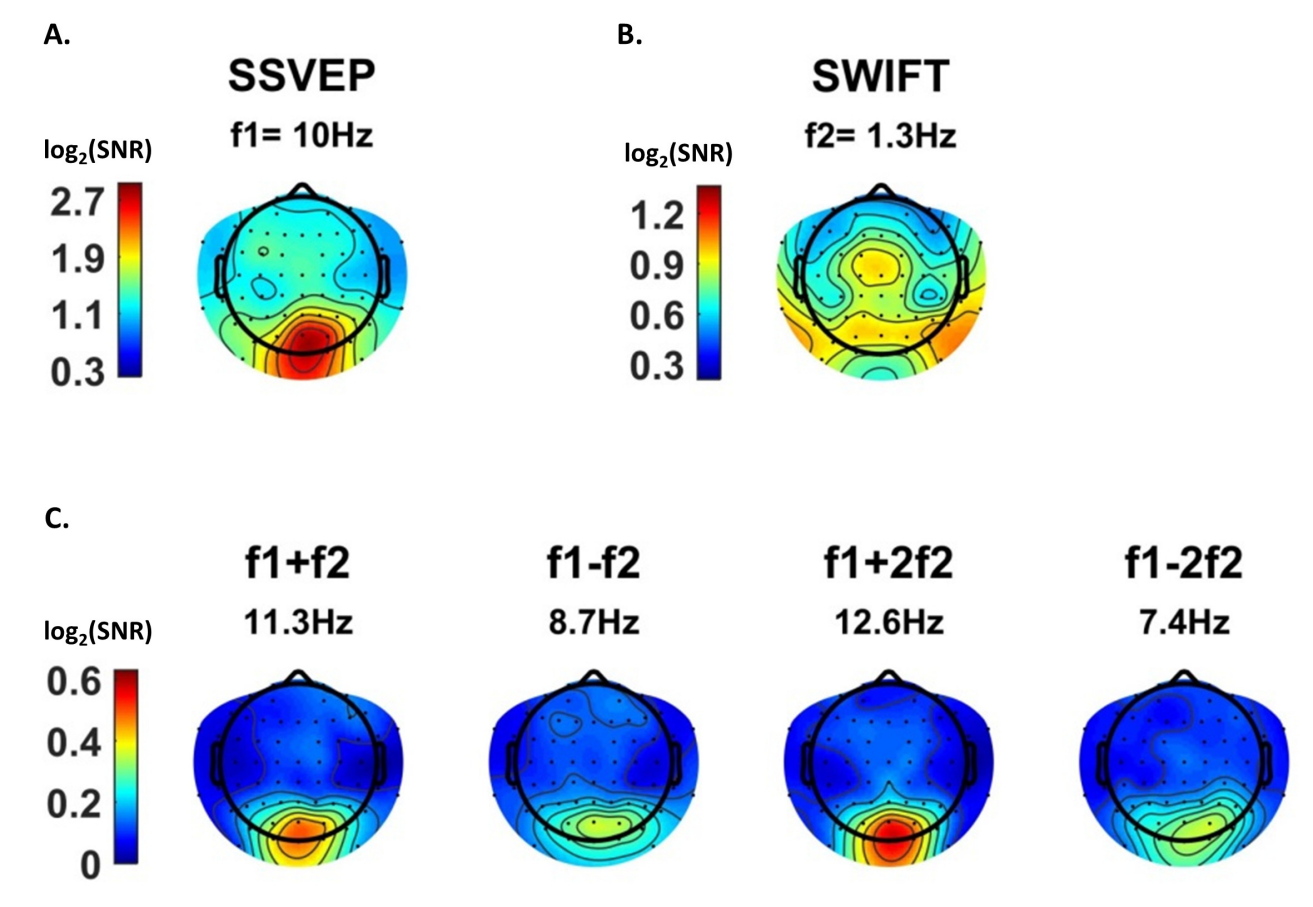
Scalp distributions. Topography maps (log2(SNR)) for SSVEP (f1 = 10 Hz) (**A**), SWIFT (f2 = 1.3 Hz) (**B**), and four IM components (f1+f2, f1−f2, f1+2f2 and f1−2f1) (**C**). SSVEP SNRs were generally stronger than SWIFT SNRs, which in turn were stronger than the IM SNRs (note the different colorbar scales). **DOI:** http://dx.doi.org/10.7554/eLife.22749.004

SSVEP amplitude signal-to-noise ratios (SNRs) were strongest, as expected, over the occipital region (Figure 3A). For SWIFT, highest SNRs were found over more temporo- and centro-parietal electrodes (Figure 3B). Strongest SNR values for the IM components were indeed found over occipital electrodes (Figure 3C). To better quantify the similarity between the scalp distributions of SSVEP, SWIFT and IM frequencies we examined the correlations between the SNR values across all 64 channels. We then examined whether the correlation coefficients for the comparison between the IMs and the SSVEP were higher than the correlation coefficients for the comparison between the IMs and the SWIFT. To do so, we applied the Fisher’s r to z transformation and performed a Z-test for the difference between correlations. We found that the distributions of all IM components were significantly more correlated with the SSVEP than with the SWIFT distribution (z = 6.44, z = 5.52, z = 6.5 and z = 6.03 for f1+f2, f1−f2, f1+2f2 and f1−2f2, respectively; two-tailed, FDR adjusted p<0.01 for all comparisons; Figure 3—figure supplement 1).

**Figure 3—figure supplement 1.**
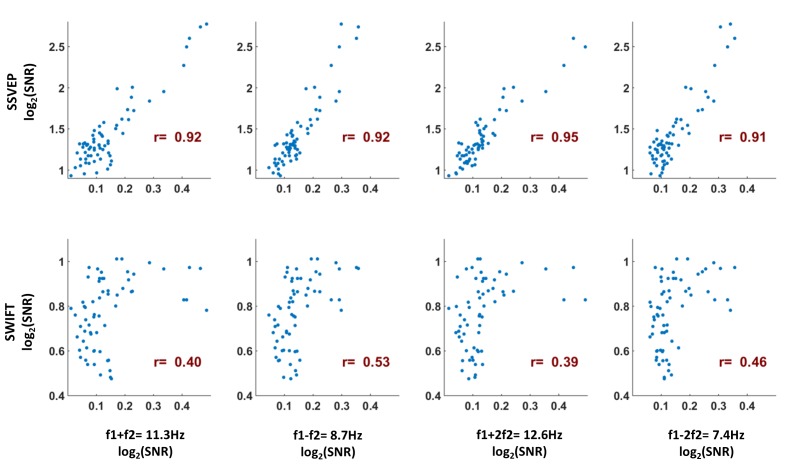
As a measure of the similarity between the scalp distributions of the SSVEP, SWIFT and IM frequencies, we examined the Pearson correlation between the mean SNR values across participants for the IM, SSVEP and SWIFT frequencies across all 64 channels (each point represents the mean SNR for a single channel across 17 participants). To examine whether the correlation coefficients for the comparison between the IMs and the SSVEP were higher than the correlation coefficients for the comparison between the IMs and the SWIFT, we applied the Fisher’s r to z transformation and performed a Z-test for the difference between correlations. We found that the distributions of all IM components were more highly correlated with the SSVEP than with the SWIFT distribution (z = 6.44, z = 5.52, z = 6.5 and z = 6.03 for f1 +f2, f1-f2, f1 +2f2 and f1-2f2, respectively; two-tailed, FDR adjusted p<0.01 for all comparisons). **DOI:** http://dx.doi.org/10.7554/eLife.22749.005

As further detailed in the Discussion, we suggest that this result is consistent with the notion that top-down signals (as tagged with SWIFT) are projected to occipital areas, where they are integrated with SSVEP-tagged signals.

The final stage of our analysis was to examine the effect of certainty on the SSVEP, SWIFT and IM signals. If the IM components observed in our data reflect a perceptual process in which bottom-up sensory signals are integrated nonlinearly with top-down predictions, we should expect them to be modulated by the level of certainty about the upcoming stimuli (here, whether the next stimulus would be a face or house image). To test this hypothesis we modulated certainty levels across trials by varying the proportion of house and face images presented.

**Figure 4.**
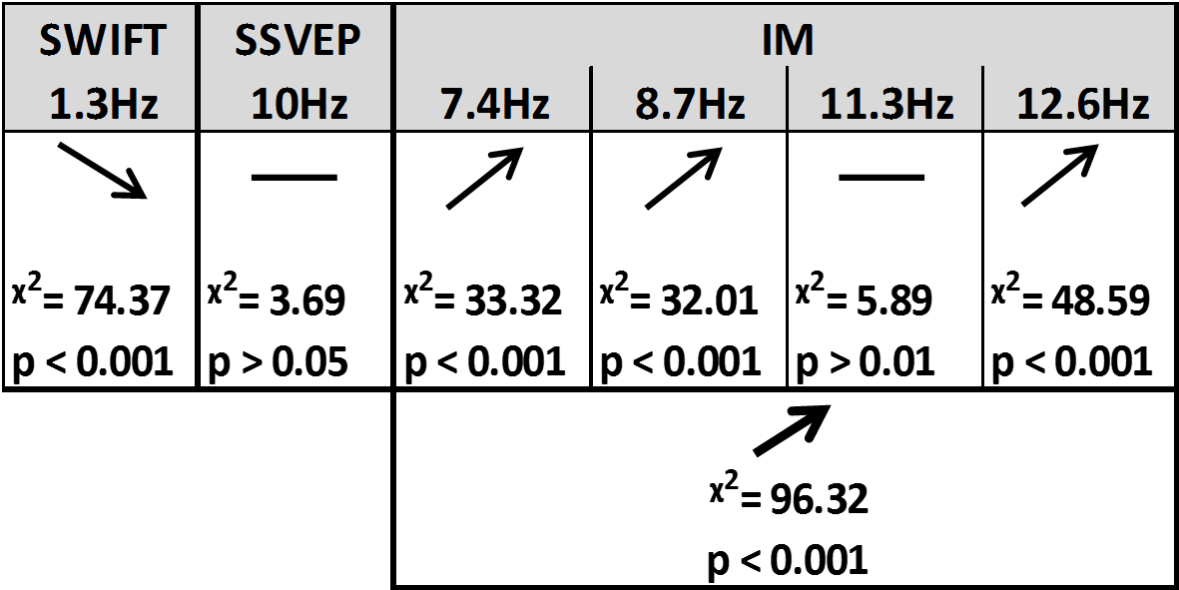
Summary of the linear mixed-effects (LME) modelling. We used LME to examine the significance of the effect of certainty for SSVEP (f1 = 10 Hz), SWIFT (f2 = 1.3 Hz) and IM (separately for f1−2f2, f1−f2, f1+f2, and f1+2f2, as well as across all four components) recorded from posterior ROI electrodes. The table lists the direction of the effects, χ2 value and FDR-corrected p-value from the likelihood ratio tests (See Materials and methods). **DOI:** http://dx.doi.org/10.7554/eLife.22749.006

**Figure 5.**
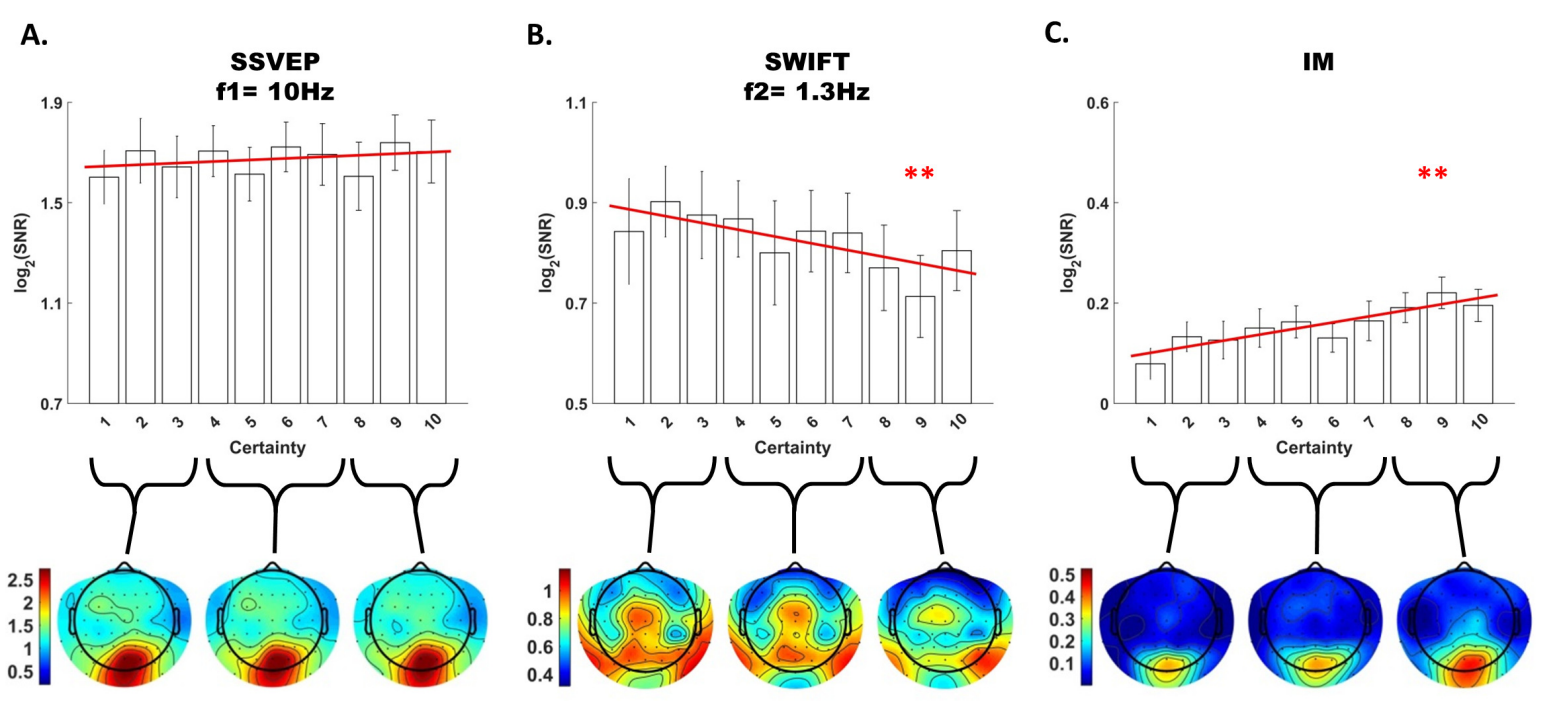
Modulation by certainty. Bar plots of signal strength (log of SNR, averaged across 30 posterior channels and 17 participants) as a function of certainty levels for SSVEP (**A**), SWIFT (**B**) and IMs (averaged across the 4 IM components) (**C**). Red lines show the linear regressions for each frequency category. Slopes that are significantly different from 0 are marked with red asterisks (** for p<0.001). While no significant main effect of certainty was found for the SSVEP (p>0.05), a significant negative slope was found for the SWIFT, and a significant positive slope was found for the IM. Error bars are SEM across participants. Bottom) Topo-plots, averaged across participants, for low certainty (averaged across bins 1–3), medium certainty (averaged across bins 4–7) and high certainty (averaged across bins 8–10) are shown for SSVEP (**A**), SWIFT (**B**) and IM (averaged across the 4 IM components) (**C**). **DOI:** http://dx.doi.org/10.7554/eLife.22749.007

Using likelihood ratio tests with linear mixed models (see Materials and methods) we found that certainty indeed had a different effect on the SSVEP, SWIFT and IM signals (Figures 4 and 5).

First, SSVEP (log of SNR at f1 = 10 Hz) was not significantly modulated by certainty (all Chi square and p-values are shown in Figure 4). This result is consistent with the interpretation of SSVEP as mainly reflecting low-level visual processing which should be mostly unaffected by the degree of certainty about the incoming signals.

Second, the SWIFT signals (log of SNR at f2 = 1.3 Hz) significantly decreased in trials with higher certainty. This is consistent with an interpretation of SWIFT as being related to the origin of top-down signals which are modulated by certainty. Specifically, better, more certain predictions would elicit less weighting for the prediction error and therefore less revisions of the high level semantic representation.

Critically, the IM signals were found to increase as a function of increasing certainty for three of the four IM components (f1−2f2 = 7.4 Hz, f1−f2 = 8.7 Hz, and f1+2f2 = 12.6 Hz though not for f1+f2 = 11.3 Hz; Figure 4). The effect remained highly significant also when including all four IM components in one model. Indeed, this is the effect we would expect to find if IMs reflect the efficacy of integration between top-down, prediction-driven signals and bottom-up sensory input. In high-certainty trials the same image appeared in the majority of cycles, allowing for the best overall correspondence between predictions and bottom-up sensory signals.

In addition, we found significant interactions between the level of certainty and the different frequency categories (SSVEP/SWIFT/IM). The certainty slope was significantly higher for the IM than for SSVEP (χ2 = 12.49, p<0.001) and significantly lower for SWIFT than for SSVEP (χ2 = 64.45, p<0.001).

## Discussion

Key to perception is the ability to integrate neural information derived from different levels of the cortical hierarchy (Fahrenfort et al., 2012; Tononi and Edelman, 1998). The goal of this study was to identify neural markers for the integration between top-down and bottom-up signals in perceptual inference, and to examine how this process is modulated by the level of certainty about the stimuli. Hierarchical Frequency Tagging combines the SSVEP and SWIFT methods that have been shown to predominantly tag low levels (V1/V2) and higher, semantically rich levels in the visual hierarchy, respectively. We hypothesised that these signals reflect bottom-up sensory-driven signals (or prediction errors) and top-down predictions. Critically, we considered intermodulation (IM) components as an indicator of integration between these signals and hypothesised that they reflect the level of integration between top-down predictions (of different strengths manipulated by certainty) and bottom-up sensory-driven input.

We found significant frequency-tagging for both the SSVEP and SWIFT signals, as well as at various IM components (Figure 2). This confirms our ability to simultaneously use two tagging methods in a single paradigm and, more importantly, provides evidence for the cortical integration of the SWIFT- and SSVEP-tagged signals. Indeed, the scalp topography for the three frequency categories (SSVEP, SWIFT and IMs) were, as we discuss further below, largely consistent with our hypotheses (Figure 3) and importantly, they all differed in the manner by which they were modulated by the level of certainty regarding upcoming stimuli. While SSVEP signals were not significantly modulated by certainty, the SWIFT signals decreased and the IM signals increased as a function of increasing certainty (Figure 5). In the following discussion we examine how our results support the predictive coding framework.

### The predictive coding framework for perception

The notion of perceptual inference and the focus on prior expectations goes back as far as Ibn al Haytham in the 11th century who noted that ‘Many visible properties are perceived by judgment and inference in addition to sensing the object’s form’ (Sabra, 1989). Contemporary accounts of perception treat these ideas in terms of Bayesian inference and predictive coding (Friston, 2005, 2009; Hohwy, 2013; Clark, 2013; Friston and Stephan, 2007). Under the predictive coding framework, hypotheses about the state of the external world are formed on the basis of prior experience. Predictions are generated from these hypotheses, which are then projected to lower levels in the cortical hierarchy, and continually tested and adjusted in light of the incoming, stimulus-driven, information. Indeed, the role of top-down signals in perception has been demonstrated in both animal and human studies (Hupe et al., 1998, Pascual-Leone and Walsh, 2001). The elements of the sensory input that cannot be explained away by the current top-down predictions are referred to as the prediction error (PE). This PE is suggested to be the (precision weighted) bottom-up signal that propagates from lower to higher levels in the cortical hierarchy until it can be explained away, allowing for subsequent revisions of higher-level parts of the overall hypotheses. The notion of PEs has been validated by numerous studies (Hughes et al., 2001; Kellermann et al., 2016; Lee and Nguyen, 2001; Todorovic et al., 2011; Wacongne et al., 2011) and several studies suggest that top-down and bottom-up signals can be differentiated in terms of their typical oscillatory frequency bands (Fontolan et al., 2014; Sedley et al., 2016; Sherman et al., 2016; Michalareas et al., 2016; Mayer et al., 2016). Perception, under the predictive coding framework, is achieved by an iterative process that singles out the hypothesis that best minimizes the overall prediction error across multiple levels of the cortical hierarchy while taking prior learning, the wider context, and precision estimations into account (Friston, 2009). Constant integration of bottom-up and top-down neural information is therefore understood to be a crucial element in perception (Fahrenfort et al., 2012; Friston, 2005; Tononi and Edelman, 1998).

### SSVEP, SWIFT and their modulation by certainty

The SSVEP method predominantly tags activity in low levels of the visual hierarchy and indeed highest SSVEP SNRs were measured in our design over occipital electrodes (Figure 3). We showed that the SSVEP signal was not significantly modulated by certainty (Figure 5A). These findings suggest that the SSVEP reflects persistent bottom-up sensory input, which does not strongly depend on top-down predictions occurring at the SWIFT frequency.

The SWIFT method, in contrast, has been shown to increasingly tag higher areas along the visual pathway which process semantic information (Koenig-Robert et al., 2015), and we indeed found highest SWIFT SNRs over more temporal and parietal electrodes (Figure 3). Since the activation of these areas depends on image recognition (Koenig-Robert and VanRullen, 2013), we hypothesised that contrary to the SSVEP, the SWIFT signal should show greater dependency on certainty. Indeed, we observed that SWIFT SNR decreased as certainty levels increased (Figure 5B).

One interpretation of this result is that it reflects the decreasing weight on PE signals under high certainty (which in turn drive the subsequent top-down predictions). The notion of certainty used here is captured well in work on the Hierarchical Gaussian Filter (Mathys et al., 2014): ‘…it makes sense that the update should be antiproportional to [the precision of the belief about the level being updated] since the more certain the agent is that it knows the true value …, the less inclined it should be to change it’ (for a mathematical formulation, see eq. 56 in that work, and, for the hierarchical case and yielding a variable learning rate, eq. 59). Indeed, various studies have previously demonstrated that highly predictable stimuli tend to evoke reduced neural responses (Alink et al., 2010; Todorovic and de Lange, 2012; Todorovic et al., 2011). Since PEs reflect the elements of sensory input that cannot be explained by predictions, such reduced neural responses have been suggested to reflect decreased PE signals (Todorovic et al., 2011).

The SWIFT SNR decline with certainty can also be described in terms of neural adaptation (or repetition suppression), that is, the reduction in the evoked neural response measured upon repetition of the same stimulus or when the stimulus is highly expected. In our current study, high-certainty trials contained more consecutive cycles in which the same image was presented, thus adaptation is expected to occur. From the predictive coding perspective, however, adaptation is explained in terms of increasing precision of predictions stemming from perceptual learning (Auksztulewicz and Friston, 2016; Friston, 2005; Henson, 2003). Adaptation then ‘reflects a reduction in perceptual 'prediction error'… that occurs when sensory evidence conforms to a more probable (previously seen), compared to a less probable (novel), percept.’ (Summerfield et al., 2008).

### Intermodulation (IM) as the marker of neural integration of top-down and bottom-up processing

The intermodulation (IM) marker was employed because studying perception requires not only distinguishing between top-down and bottom-up signals but also examining the integration between such signals. Accordingly, the strength of the Hierarchical Frequency Tagging (HFT) paradigm is in its potential ability to obtain, through the occurrence of IM, a direct electrophysiological measure of integration between signals derived from different levels in the cortical hierarchy.

From the most general perspective, the presence of IM components simply imply a non-linear integration of the steady-state responses elicited by the SWIFT and SSVEP manipulations. Various biologically plausible neural circuits for implementing nonlinear neuronal operations have been suggested (Kouh and Poggio, 2008), and such non-linear neuronal dynamics may be consistent with a number of models, ranging from cascades of non-linear forward filters (e.g., convolution networks used in deep learning) through to the recurrent architectures implied by predictive coding. The presence of IMs in themselves therefore cannot point conclusively at specific computational or neuronal processes to which the IMs could be mapped. Suggesting IMs as evidence for predictive coding rather than other theories of perception therefore remains to some degree indirect, however, various arguments indeed point to the recurrent and top-down mediation of the IM responses in our data.

First, the scalp distributions of the IM components were more strongly correlated to the spatial distribution of the SSVEP (f1 = 10 Hz) rather than to the SWIFT (f2 = 1.3 Hz) (Figure 3—figure supplement 1). This pattern supports the notion that the IM components in our Hierarchical Frequency Tagging (HFT) data reflect the integration of signals generated in SWIFT-tagged areas which project to, and are integrated with, signals generated at lower levels of the visual cortex, as tagged by the SSVEP. This of course is consistent with the predictive coding framework in which predictions generated at higher levels in the cortical hierarchy propagate to lower areas in the hierarchy where they can be tested in light of incoming sensory-driven signals.

Second, and more importantly, the IM SNRs increased as a function of certainty (contrary to the SWIFT SNR). We suggest that this result lends specific support to the predictive coding framework where translating predictions into prediction errors rests upon nonlinear functions (Auksztulewicz and Friston, 2016). Indeed, nonlinearities in predictive coding models are a *specific* corollary of top-down modulatory signals (Friston, 2005). Varying certainty levels, as operationalised in our stimuli, would therefore be expected to impact IM signal strength through the nonlinear modulation of bottom-up input by top-down predictions. Specifically, higher certainty trials induced greater predictability of upcoming images and a greater overall match throughout the trial between predictions and sensory input. The increase in IM SNRs in our data may therefore reflect the efficient integration of, or the overall ‘fit’ between, predictions and sensory input that should be expected when much of the upcoming stimuli is highly predictable.

### Mapping HFT responses to predictive coding models

In line with the notion above, it is possible to suggest a more specific mapping of the HFT components (SWIFT, SSVEP and IMs) onto elements of predictive coding. According to the model set forward by Auksztulewicz and Friston (Auksztulewicz and Friston, 2016), for example, top-down nonlinearities (functions g and f in equations 6 and 7, as well as in Figure 1 in that work) are driven by two elements: (1) the conditional expectations of the hidden causes (µ_v_, i.e. the brain’s ‘best estimate’ as to what is driving the changes in the physical world), and (2) the conditional expectations of the hidden states (µ_x_, i.e. the brain’s best estimate about the actual ‘physics’ of the external world that drives the responses of the sensory organs). The relationships between possible ‘causes’ and ‘states’ (e.g. how the movement of a cloud in the sky impacts the luminance of objects on the ground) is learnt over time and is the crux of the dynamic generative model embodied by the brain. Appealing to this model, the conditional expectations of hidden causes and states may be suggested to be driven primarily by the SWIFT (tagging activity in areas rich in semantic information) and the SSVEP (tagging activity in areas responding to low-level visual features), respectively. Top-down predictions can therefore be expected to result in the formation of the IM components that reflect the nonlinear integration of SWIFT- and SSVEP-driven signals.

A further question concerns potential quantitative interpretations of the IMs and their increase with certainty. One such interpretation is that the IMs collectively encode (some approximation to the log) model evidence. This notion is compatible with our interpretation of IMs in terms of the ‘fit’ between predictions and sensory input. In this case, one would expect the IMs to increase with certainty, as shown in Figure 5c. It is an interesting question for further research if this interpretation of IM as encoding model evidence can generate quantitative predictions for the IM magnitude in different experimental manipulations of SWIFT and SSVEP, and further, if different IMs might result from distinct manipulations of expectations and precisions.

### Alternative interpretations for the IM components

One could potentially argue that our IM findings may arise from sensory processing alone. For example, consider a population of neurons confined within the visual cortex, in which some are modulated by stimulus contrast via SSVEP and some are modulated by category information via SWIFT. Interactions between these neurons, in such an essentially feedforward mechanism, may potentially account for the formation of IM components even without any top-down signals. However, this alternative interpretation cannot easily account for the pattern of reciprocal changes with certainty found in our data (decreasing SWIFT and increasing IMs). Integration of bottom-up sensory input alone should be blind to the probabilistic properties of the trial such that accounting for the pattern of data here requires suggesting an additional local mechanism which is sensitive to the certainty manipulation. Therefore, it seems more reasonable to assume an interaction between early and higher sensory areas, which have been shown to be sensitive to the predictability of stimuli (Kok et al., 2012a, Rauss et al., 2011).

In addition, the IM components could in principle result from the integration of low-level SSVEP signals with minimal, non-semantic, SWIFT-driven signals entrained in the early visual cortex (e.g. by residual tagging of the noise components within the SWIFT frames). While this possibility cannot be fully excluded, previous findings suggest that SWIFT does not tag V1-level activity as no tagging could be detected neither for trials in which non-semantic patterns were used nor for trials in which attention was driven away from the image (Koenig-Robert and VanRullen, 2013; Koenig-Robert et al., 2015). Residual low-level SWIFT-tagging is therefore not likely to be the primary contributor to the IM components found here.

Several studies have demonstrated a relationship between IM components and perception (Boremanse et al., 2013; Gundlach and Müller, 2013; Zhang et al., 2011). In all of these studies, the reported increase in IM signal strength potentially reflects the integration of different input elements within a single neural representation. However, the strength of Hierarchical Frequency Tagging is in its ability to simultaneously tag both bottom-up *and* top-down inputs to the lower visual areas. The IM signals, in our paradigm, would then reflect the crux of the hypothesis-testing function, namely, the comparison of prediction and sensory-driven signals, or the integration between state-units and error-units.

### Manipulating certainty through implicit learning

An additional point worth noting is that the certainty manipulation we used in this study differs from several other studies (e.g. [Kok et al., 2013; Kok et al., 2012a]) whereby expectation is explicitly manipulated with a preceding cue. In each of the current study’s trials certainty levels were learnt ‘online’ based on the proportion of images that appeared in that trial. Operationalizing certainty in this manner may add sources of variability we did not control for, such as individual differences in learning rates. On the other hand, belief about the probability of an event is often shaped through repeated exposure to the same type of event, placing greater ecological validity to our study design. It is an interesting question for further research whether a priori knowledge of certainty levels will give rise to different IMs, as well as whether individual differences in learning rates (including for example differences in ‘optimal forgetting’, [Mathys et al., 2014]) affect IMs.

### Conclusion

Overall, the evidence we have presented plausibly demonstrates the ability of the novel HFT technique to obtain a direct physiological measure of the integration of information derived from different levels of the cortical hierarchy during perception. Supporting the predictive coding account of perception, our results suggest that top-down, semantically tagged signals are integrated with bottom-up sensory-driven signals, and this integration is modulated by the level of certainty about the causes of the perceived input.

## Materials and methods

### Stimulus construction

#### SSVEP and SWIFT

In steady-state-visual-evoked-potentials (SSVEP) studies, the intensity (luminance or contrast) of a stimulus is typically modulated over time at a given frequency, *F* Hz (i.e. the ‘tagging frequency’). Peaks at the tagging-frequency, *f* Hz, in the spectrum of the recorded signal are thus understood to reflect stimulus-driven neural activity. However, the use of SSVEP methods impose certain limitations for studying perceptual hierarchies. When the contrast or luminance of a stimulus is modulated over time, then all levels of the visual hierarchy are entrained at the tagging frequency. Thus, it becomes difficult to dissociate frequency tagging related to low-level feature processing from that related to high-level semantic representations.

Semantic wavelet-induced frequency-tagging (SWIFT) overcomes this obstacle by scrambling image sequences in a way that maintains low-level physical features while modulating mid to high-level image properties. In this manner, SWIFT has been shown to constantly activate early visual areas while selectively tagging high-level object representations both in EEG (Koenig-Robert and VanRullen, 2013) and fMRI (Koenig-Robert et al., 2015).

The method for creating the SWIFT sequences is described in detail elsewhere (Koenig-Robert and VanRullen, 2013). In brief, sequences were created by cyclic wavelet scrambling in the wavelets 3D space, allowing to scramble contours while conserving local low-level attributes such as luminance, contrast and spatial frequency. First, wavelet transforms were applied based on the discrete Meyer wavelet and six decomposition levels. At each location and scale, the local contour is represented by a 3D vector. Vectors pointing at different directions but of the same length as the original vector represent differently oriented versions of the same local image contour. Two such additional vectors were randomly selected in order to define a circular path (maintaining vector length along the path). The cyclic wavelet-scrambling was then performed by rotating each original vector along the circular path. The inverse wavelet transform was then used to obtain the image sequences in the pixel domain. By construction, the original unscrambled image appeared once in each cycle (1.3 Hz). The original image was identifiable briefly around the peak of the embedded image (see Video 1, also available at https://figshare.com/s/44f1a26ecf55b6a35b2f), as has been demonstrated psychophysically (Koenig-Robert et al., 2015).

**Video 1.** A slow-motion representation of two SWIFT cycles. **DOI:** http://dx.doi.org/10.7554/eLife.22749.008

#### SWIFT-SSVEP trial

SWIFT sequences were created from a pool of grayscale images of houses and faces (28 each, downloaded from the Internet using Google Images (https://www. google.com/imghp) to find images with ‘free to use, share or modify, even commercially’ usage rights; Figure 1A–B).

Each trial was constructed using one house and one face sequence, randomly selected from the pool of sequences (independently from the other trials). Using these two sequences, which, in the context of a full trial we refer to as SWIFT ‘cycles’, we created a 50 s ‘movie’ containing 65 consecutive cycles repeated in a pseudorandom order at F2 = 1.3 Hz (~769 ms per cycle, Figure 1D). The identifiable image at the peak of each cycle was either the face or the house image. The SWIFT method was designed to ensure that the low-level local visual properties within each sequence (cycle) are preserved across all frames. However, these properties could differ significantly between the face and the house sequences, resulting in the potential association of SWIFT-tagged activity with differences in the low level features between the face and house cycles. To prevent this, we created and merged additional ‘noise’ sequences in the following way: First, we selected one of the scrambled frames from each of the original SWIFT sequences (the ‘most scrambled’ one, i.e. the frame most distant from the original image presented at the peak of the cycle). Then, we created noise sequences by applying the SWIFT method on each of the selected scrambled frames. In this way, each original ‘image’ sequence had a corresponding ‘noise’ sequence that matched the low-level properties of the image sequence. Finally, ‘image’ sequences were alpha blended with the ‘noise’ sequences of the other category with equal weights (Figure 1C, image sequences are surrounded by solid squares and noise sequences with dashed squares). For example, cycles in which a face image was to appear contained the face image sequence superimposed with a house noise sequence (Figure 1C, right side). This way, the overall low level visual attributes were constant across all frames in the trial regardless of the identifiable image in each cycle.

A global sinusoidal contrast modulation at F1 = 10 Hz was applied on the whole movie to evoke the SSVEP (see Videos 2 and 3, also available at https://figshare.com/s/75aed271d32ba024d1ee).

**Video 2.** An 8 s animated movie representation of a HFT trial. **DOI:** http://dx.doi.org/10.7554/eLife.22749.009

**Video 3.** A slow motion animation of the first few cycles within a HFT trial. **DOI:** http://dx.doi.org/10.7554/eLife.22749.010

### Participants and procedure

A total of 27 participants were tested for this study (12 females; mean age = 28.9 y, std = 6.6). Participants gave their written consent to participate in the experiment. Typical sample sizes in SSVEP and SWIFT studies range between 8–22 participants per experimental group (Chicherov and Herzog, 2015; Katyal et al., 2016; Koenig-Robert and VanRullen, 2013; Koenig-Robert et al., 2015; Painter et al., 2014). As this is the first study to simultaneously combine the SWIFT and SSVEP tagging methods we aimed to be on the higher end of this range. Experimental procedures were approved by the Monash University Human Research Ethics Committee.

Participants were comfortably seated with their head supported by a chin rest 50 cm from the screen (CRT, 120 HZ refresh rate) in a dimly lit room. Sequences were presented at the center of the screen over a grey background and participants were asked to keep their fixation at the center of the display. Participants were asked to minimise blinking or moving during each trial, but were encouraged to do so if needed in the breaks between each 50 s trial. A total of 56 such 50 s trials were presented to each participant. Importantly, the proportion of house and face images varied over trials, spanning the full possible range (pseudorandomly selected such that a particular proportion was not repeated within each participant). Each trial therefore varied in the level of certainty associated with upcoming images.

In order to verify that the participants engaged with the task, a sentence appeared on the screen before each trial instructing them to count either the number of house or face presentations. Trials began when the participant pressed the spacebar. They used the keyboard at the end of each trial to enter the number of images counted. These responses were recorded and used later to exclude poorly-performing participants from the analysis. A 2–3 min rest break was introduced after every 14 trials. Continuous EEG was acquired from 64 scalp electrodes using a Brain Products BrainAmp DC system. Data were sampled at 1000 Hz for 23 participants and at 500 Hz for the remaining four participants.

### Data analysis

Data processing was performed using the EEGLAB toolbox (Delorme and Makeig, 2004) in MATLAB. All data sampled at 1000 Hz were resampled to 500 Hz. A high-pass filter was applied at 0.6 Hz and data was converted to average reference.

#### Exclusion criteria

**Figure 6.**
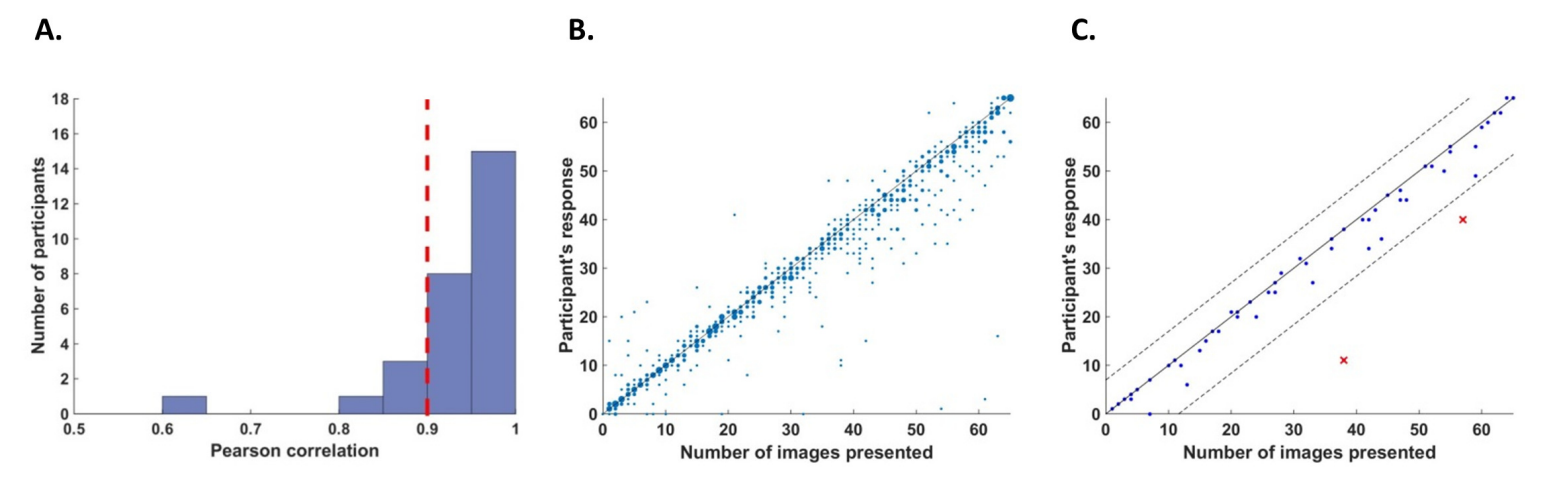
Behavioral performance. (**A**) Histogram across all participants for counting accuracy measured as the correlation between the participant’s response (number of image presentations counted in each trial) and the actual number of presentations. Five participants with a counting accuracy below r = 0.9 (vertical red dashed line) were excluded from the analysis. (**B**) Scatter plot showing responses across 56 trials for all participants included in the analysis. The size of each dot corresponds to the number of occurrences at that point. (**C**) An example scatter plot for a single participant demonstrating the within-participant exclusion criterion for single trials. The solid line (y=x) illustrates the theoretical location of accurate responses. For each trial, we calculated the distance between the participant’s response and the actual number of cycles in which the relevant image was presented (i.e., the distance between each dot in the plot and the solid line). The within-participant cutoff was then defined as ±2.5 standard deviations from the mean of this distance. Dashed lines mark the within-participant cutoff for exclusion of single trials. **DOI:** http://dx.doi.org/10.7554/eLife.22749.011

We defined two criteria to exclude participants from the analysis. First, we excluded participants who had poor counting accuracy because we cannot be sure if these participants were attentive throughout the task. For this purpose, we calculated correlations for each participant between their responses (number of image presentations counted in each trial) and the actual number of cycles in which the relevant image was presented. We excluded five participants whose correlation value r was lower than 0.9 (Figure 6A).

The second criterion was based on the quality of EEG recordings. Sample points were regarded as being noisy if they were either greater than ±80 μV, contained a sudden fluctuation greater than 40μV from the previous sample point, or if the signal was more than ±6 std from the mean of the trial data in each channel. Cycles in which over 2% of sample points were noisy were regarded as noisy cycles. For each channel, all sample points within the noisy cycles were replaced by the mean signal across the trial. Participants for which over 10% of cycles were noisy were excluded from the analysis. Five additional participants were excluded on the basis of this criterion for poor EEG recording (on average, 37% of cycles were noisy for these participants). A total of 17 remaining participants were included in the analysis.

In addition, we excluded within-participant subsets of trials. For each participant, we calculated the mean and standard deviation of the difference between the participant’s response (count) and the number of cycles in which the relevant image was presented. We then excluded all trials in which the participant’s response fell further than 2.5 standard deviations from his mean accuracy (e.g., Figure 6C). From this criterion, we excluded 5.5% of the trials (52 out of 952 trials in total, 0–5 trials out of 56 for any individual participant).

#### Spectral analysis

EEG signal amplitude was extracted at the tagging and intermodulation frequencies by applying the fast Fourier transform (FFT) over each trial (50 s, 25,000 sample-points, frequency resolution = 0.02 Hz). Signal-to-noise ratios (SNR) at frequency f was computed by dividing the amplitude at f by the mean amplitude across 20 neighbouring frequencies (from f-0.2Hz to f-0.02Hz and from f + 0.02 Hz to f + 0.2 Hz) (Srinivasan et al., 1999; Tononi and Edelman, 1998).

##### Intermodulation components

IM components include all linear combinations of the fundamental frequencies that comprise the input signal (n1f1 + n2f2, n = ±1,±2,±3…). While a large number of potential IM components exist in our data, we focused our analysis on the four lowest-order components (f1−2f2 = 7.4 Hz, f1−f2 = 8.7 Hz, f1+f2 = 11.3 Hz and f1+2f2 = 12.6 Hz, where f1 = 10 Hz and f2 = 1.3 Hz).

#### Statistical analysis

For analysis of the modulatory effects of certainty we used RStudio (RStudio Team, 2015) and lme4 (Bates et al., 2015) to perform linear mixed-effect analysis of the data. Eight frequencies of interest were analysed: f2 = 1.3 Hz and 2f2 = 2.6 Hz (SWIFT and harmonic), f1 = 10 Hz and 2f1 = 20 Hz (SSVEP and harmonic), and f1-2f2 = 7.4 Hz, f1−f2 = 8.7 Hz, f1+f2 = 11.3 Hz and f1+2f2 = 12.6 Hz (IM components). We used log_2_(amplitude SNR) as the dependant variable for all analyses. We chose this transformation because the amplitude SNR has a lower bound of 0 and does not distribute normally. The distribution of log_2_(SNR) on the other hand is closer to a normal distribution and allows for better homoscedasticity in the linear models.

In order to examine the modulatory effect of certainty, we divided trials into 10 certainty bins ranging from 1 (lowest certainty) to 10 (highest certainty). Bin limits were defined in terms of the percentage of cycles at which the more frequent image appeared, thus creating 5%-wide bins (trials in which the more frequent image appeared in 50–55%, 55–60%, … and 95–100% of cycles are defined as bin 1, 2, ... and 10, respectively).

Different statistical models were applied for each of the three levels of analysis performed: (1) within each of 6 frequencies of interest (e.g., f1, f2, f1+f2, etc.), (2) within the IM category (f1−2f2, f1−f2, f1+f2 and f1+2f2) and (3) between frequency categories (SSVEP/SWIFT/IM). All analyses were performed on a posterior ROI (30 electrodes) including all centro-parietal (CPz and CP1-CP6), temporo-parietal (TP7-TP10), parietal (Pz and P1-P8), parieto-occipital (POz, PO3-PO4, and PO7-PO10) and occipital (Oz,O1 and O2) electrodes. Channels were added to all models as a random effect. All random effects allowed for both random intercepts and slopes.

To examine if certainty had a significant modulatory effect within each frequency of interest, the first level of analysis included certainty as the fixed effect, and channel nested within participants as the random effect. To examine if there was a main effect for certainty within each frequency category (SSVEP/SWIFT/IM), the second level of analysis included certainty as the fixed effect, and frequency nested within channel nested within participants as the random effect. To examine if the main effect of certainty differed between frequency categories (i.e. a significant interaction between certainty and frequency category), the third level of analysis included certainty, frequency category and a certainty-category interaction as the fixed effects, and frequency nested within frequency category nested within channel nested within participants as the random effect.

To test for the significance of a given factor or interaction, we performed likelihood ratio tests between the full model, as described above, and the reduced model which did not include the factor or interaction in question (Bates et al., 2015). When applicable, we adjusted p values using the false discovery rate (Yekutieli and Benjamini, 1999).
