# Supplementary material for: Neural markers of predictive coding under perceptual uncertainty revealed with Hierarchical Frequency Tagging
Source: eLife. 2017 Feb 28;6:e22749. doi: 10.7554/eLife.22749 (PMC5360443; doi:10.7554/eLife.22749)
Supplement: Related texts

# Article commentary: Decision letter

**DOI:** 10.7554/eLife.22749.012

**Affiliations:** University of Zurich and ETH Zurich, Switzerland

In the interests of transparency, eLife includes the editorial decision letter and accompanying author responses. A lightly edited version of the letter sent to the authors after peer review is shown, indicating the most substantive concerns; minor comments are not usually included.

Thank you for submitting your article "Neural markers of predictive coding under perceptual uncertainty revealed with Hierarchical Frequency Tagging" for consideration by *eLife*. Your article has been favorably evaluated by Timothy Behrens (Senior Editor) and three reviewers, one of whom, Klaas Enno Stephan (Reviewer #1), is a member of our Board of Reviewing Editors. The following individual involved in review of your submission has agreed to reveal their identity: Peter Kok (Reviewer #2).

The reviewers have discussed the reviews with one another and the Reviewing Editor has drafted this decision to help you prepare a revised submission.

Summary of manuscript:

This EEG study of healthy human volunteers tests a key corollary of predictive coding: that brain activity of visual areas during perceptual inference should reflect the integration of top-down predictions with bottom-up inputs. To test this hypothesis, this study uses a novel hierarchical frequency tagging method which combines a recently developed semantic wavelet induced frequency tagging (SWIFT) with classical SSVEP (here: a sinusoidal contrast modulation of images). This allows for studying the effects of predictions and sensory inputs on brain activity in separate frequency bands and, importantly, examine integration of both signals in terms of intermodulation components.

Summary of reviews:

Overall, all reviewers agreed that the paper contributes an important step forward in empirical investigations of predictive coding. In particular, all three reviewers liked the experimental approach adopted by this study and agreed that it represents a novel and clever method to test a central aspect of predictive coding theory. We had an engaging discussion (and slightly diverging opinions) about potential limits of interpretation due to experimental confounds; this is detailed below and represents a key issue that needs to be addressed convincingly in the revision of the manuscript.

The policy of the journal is to provide you with a single set of comments which reflect the consensus view amongst reviewers. These comments can be found below and must be addressed convincingly. We hope that you will find these comments helpful to further improve the paper.

Essential revisions:

1) IM components are said to reflect integration of predictions and sensory inputs, but could they be the result of sensory inputs alone? That is, if both SSVEP and SWIFT components are present in the occipital cortex (the fact that the scalp distribution for SWIFT also seems to include some more anterior sensors does not mean that these signals are absent in the occipital cortex), we wondered whether the IMs might also be present there as a result of sensory processing. To give an intuition for this: if certain neurons respond to, let's say, houses, and if their 'house response' is modulated by the contrast of the house stimulus (as is highly likely), wouldn't these neurons show IM components without any involvement of top-down predictions? This point requires, at the very least, detailed consideration in the Discussion. It could potentially be tested by source space analyses, examining whether responses in FFA/OFA and PPA, respectively, show interactions between semantic category and contrast. However, we appreciate that this is a non-trivial extension of the study and leave the decision to the authors.

2) We wondered to what degree the effects of certainty on SWIFT components might reflect adaptation effects. This is because under high certainty, consecutive images are more likely to be of the same category, thus possibly leading to adaptation in face/house sensitive neurons and hence a reduced SWIFT response. In other words, in the present design, certainty is confounded with stimulus adaptation. Again, this point would require a detailed discussion and the limits of interpretation should be acknowledged clearly.

3) We had an extensive discussion about the interpretation of the IM responses and their increase with certainty in Figure 4C in computational (Bayesian) and neurophysiological terms. There was some concern that it is difficult to interpret this slope in Figure 4C unless one proposes a particular neuronal model that implements predictive coding and emits the IMs observed here. To illustrate the range of possible interpretations, two possibilities may be worth considering: If these IMs (collectively) represented the expectation of posterior beliefs, one might expect them to decline with certainty because "certainty" in this study equals the precision of prediction which should downweight prediction errors / reduce synaptic gain (in contradistinction to precision of sensory input) in predictive coding. If, however, these IMs encoded (some approximation to the log) model evidence – a notion compatible with the argument put forward by the authors – one would expect them to increase with certainty, as shown in the plot.

4) A general point that deserves more detailed discussion is that using IM components as evidence for the operation of nonlinear integration has pros and cons. The beauty is that this circumvents the need to choose a particular implementation of predictive coding. The downside is that the absence of such a neuronal model renders it impossible to map distinct IM components to specific computational or neuronal processes. IMs simply provide evidence for a nonlinear interaction between different (experimentally controlled) frequencies. While this is indeed a corollary of predictive coding, it does not appear to be a specific one and may also emerge from other theories of perceptual inference.

# Reply: Author response

**DOI:** 10.7554/eLife.22749.013

*Essential revisions:*

*1) IM components are said to reflect integration of predictions and sensory inputs, but could they be the result of sensory inputs alone? That is, if both SSVEP and SWIFT components are present in the occipital cortex (the fact that the scalp distribution for SWIFT also seems to include some more anterior sensors does not mean that these signals are absent in the occipital cortex), we wondered whether the IMs might also be present there as a result of sensory processing. To give an intuition for this: if certain neurons respond to, let's say, houses, and if their 'house response' is modulated by the contrast of the house stimulus (as is highly likely), wouldn't these neurons show IM components without any involvement of top-down predictions? This point requires, at the very least, detailed consideration in the Discussion. It could potentially be tested by source space analyses, examining whether responses in FFA/OFA and PPA, respectively, show interactions between semantic category and contrast. However, we appreciate that this is a non-trivial extension of the study and leave the decision to the authors.*

Thank you for this important comment. The alternative mechanism of neuronal integration involving sensory inputs alone should indeed be considered and addressed in the paper. We have given this issue much thought and have revised, employing your comments, to reflect that there are different ways to formulate this alternative interpretation. We also considered the possibility of source analysis, which we believe is an important avenue of research on IMs. We are currently thinking of the best study design (e.g., use of MEG rather than EEG for better localization) using hierarchical frequency tagging for source localization analysis. However, we believe that this may best be done in a separate future paper.

To address the alternative interpretation, we have revised our Discussion by inserting a subsection titled “Alternative interpretations for the IM components”:

“One could potentially argue that our IM findings may arise from sensory processing alone. […] Residual low-level SWIFT-tagging is therefore not likely to be the primary contributor to the IM components found here.”

*2) We wondered to what degree the effects of certainty on SWIFT components might reflect adaptation effects. This is because under high certainty, consecutive images are more likely to be of the same category, thus possibly leading to adaptation in face/house sensitive neurons and hence a reduced SWIFT response. In other words, in the present design, certainty is confounded with stimulus adaptation. Again, this point would require a detailed discussion and the limits of interpretation should be acknowledged clearly.*

We agree this is an important point to consider in the paper. We have changed the manuscript to discuss this point and acknowledge this as a potential confound. Overall, we think this actually gives a meaningful and interesting interpretation of our results, namely in terms of predictive coding approaches to adaptation. We have inserted the following paragraph in Discussion:

“The SWIFT SNR decline with certainty can also be described in terms of neural adaptation (or repetition suppression), that is, the reduction in the evoked neural response measured upon repetition of the same stimulus or when the stimulus is highly expected. […] Adaptation then “reflects a reduction in perceptual 'prediction error'… that occurs when sensory evidence conforms to a more probable (previously seen), compared to a less probable (novel), percept.” (Summerfield et al., 2008).”

*3) We had an extensive discussion about the interpretation of the IM responses and their increase with certainty in Figure 4C in computational (Bayesian) and neurophysiological terms. There was some concern that it is difficult to interpret this slope in Figure 4C unless one proposes a particular neuronal model that implements predictive coding and emits the IMs observed here. To illustrate the range of possible interpretations, two possibilities may be worth considering: If these IMs (collectively) represented the expectation of posterior beliefs, one might expect them to decline with certainty because "certainty" in this study equals the precision of prediction which should downweight prediction errors / reduce synaptic gain (in contradistinction to precision of sensory input) in predictive coding. If, however, these IMs encoded (some approximation to the log) model evidence – a notion compatible with the argument put forward by the authors – one would expect them to increase with certainty, as shown in the plot.*

Thank you for this helpful comment; we have welcomed this challenge and have added a subsection in the Discussion titled “Mapping HFT components to predictive coding models”. We believe that this provides the interested reader with a more in depth discussion about the possible links to specific elements of predictive coding (both in terms of sources of nonlinearities and in terms of quantitative interpretations of the IMs):

“In line with the notion above, it is possible to suggest a more specific mapping of the HFT components (SWIFT, SSVEP and IMs) onto elements of predictive coding. […] It is an interesting question for further research if this interpretation of IM as encoding model evidence can generate quantitative predictions for the IM magnitude in different experimental manipulations of SWIFT and SSVEP, and further, if different IMs might result from distinct manipulations of expectations and precisions.”

Note: Given the arrows in Table 1, *eLife*’s editorial suggested that it might be better to process this as an image. We have accordingly labelled the table as ‘Figure 4’ and as a result, the original Figures 4 and 5 are now labelled Figures 5 and 6, respectively.

*4) A general point that deserves more detailed discussion is that using IM components as evidence for the operation of nonlinear integration has pros and cons. The beauty is that this circumvents the need to choose a particular implementation of predictive coding. The downside is that the absence of such a neuronal model renders it impossible to map distinct IM components to specific computational or neuronal processes. IMs simply provide evidence for a nonlinear interaction between different (experimentally controlled) frequencies. While this is indeed a corollary of predictive coding, it does not appear to be a specific one and may also emerge from other theories of perceptual inference.*

We acknowledge that this is indeed a strength of the study but also a limitation. We have indicated our answer below, which also to some extent depends on our answers to point 1 and 3 above. We have inserted the following paragraph in the Discussion:

“From the most general perspective, the presence of IM components simply imply a non-linear integration of the steady-state responses elicited by the SWIFT and SSVEP manipulations. […] Suggesting IMs as evidence for predictive coding rather than other theories of perception therefore remains to some degree indirect, however, various arguments indeed point to the recurrent and top-down mediation of the IM responses in our data…”
